# Putting PrEP into Practice: Lessons Learned from Early-Adopting U.S. Providers’ Firsthand Experiences Providing HIV Pre-Exposure Prophylaxis and Associated Care

**DOI:** 10.1371/journal.pone.0157324

**Published:** 2016-06-15

**Authors:** Sarah K. Calabrese, Manya Magnus, Kenneth H. Mayer, Douglas S. Krakower, Adam I. Eldahan, Lauren A. Gaston Hawkins, Nathan B. Hansen, Trace S. Kershaw, Kristen Underhill, Joseph R. Betancourt, John F. Dovidio

**Affiliations:** 1 Department of Chronic Disease Epidemiology, Yale School of Public Health, Yale University, New Haven, CT, United States of America; 2 Center for Interdisciplinary Research on AIDS, Yale University, New Haven, CT, United States of America; 3 Department of Epidemiology and Biostatistics, Milken Institute School of Public Health, George Washington University, Washington, DC, United States of America; 4 The Fenway Institute, Fenway Health, Boston, MA, United States of America; 5 Beth Israel Deaconess Medical Center, Harvard Medical School, Boston, MA, United States of America; 6 Department of Health Promotion and Behavior, College of Public Health, University of Georgia, Athens, GA, United States of America; 7 Yale Law School, Yale University, New Haven, CT, United States of America; 8 Disparities Solutions Center, Massachusetts General Hospital, Harvard Medical School, Boston, MA, United States of America; 9 Department of Psychology, Yale University, New Haven, CT, United States of America; Curtin University, AUSTRALIA

## Abstract

Optimizing access to HIV pre-exposure prophylaxis (PrEP), an evidence-based HIV prevention resource, requires expanding healthcare providers’ adoption of PrEP into clinical practice. This qualitative study explored PrEP providers’ firsthand experiences relative to six commonly-cited barriers to prescription—financial coverage, implementation logistics, eligibility determination, adherence concerns, side effects, and anticipated behavior change (risk compensation)—as well as their recommendations for training PrEP-inexperienced providers. U.S.-based PrEP providers were recruited via direct outreach and referral from colleagues and other participants (2014–2015). One-on-one interviews were conducted in person or by phone, transcribed, and analyzed. The sample (*n* = 18) primarily practiced in the Northeastern (67%) or Southern (22%) U.S. Nearly all (94%) were medical doctors (MDs), most of whom self-identified as infectious disease specialists. Prior experience prescribing PrEP ranged from 2 to 325 patients. Overall, providers reported favorable experiences with PrEP implementation and indicated that commonly anticipated problems were minimal or manageable. PrEP was covered via insurance or other programs for most patients; however, pre-authorization requirements, laboratory/service provision costs, and high deductibles sometimes presented challenges. Various models of PrEP care and coordination with other providers were utilized, with several providers highlighting the value of clinical staff support. Eligibility was determined through joint decision-making with patients; CDC guidelines were commonly referenced but not considered absolute. Patient adherence was variable, with particularly strong adherence noted among patients who had actively sought PrEP (self-referred). Providers observed minimal adverse effects or increases in risk behavior. However, they identified several barriers with respect to accessing and engaging PrEP candidates. Providers offered a wide range of suggestions regarding content, strategy, and logistics surrounding PrEP training, highlighting sexual history-taking and sexual minority competence as areas to prioritize. These insights from early-adopting PrEP providers may facilitate adoption of PrEP into clinical practice by PrEP-inexperienced providers, thereby improving access for individuals at risk for HIV.

## Introduction

Approximately 45,000 Americans are newly infected with HIV every year [1], highlighting the need to expand the menu of prevention options routinely offered to at-risk individuals in medical care. Oral antiretroviral pre-exposure prophylaxis (PrEP) has been shown to be a well-tolerated and effective method of protection in multiple clinical trials and demonstration projects [2] and should be included among the health services provided to patients at risk for HIV due to sexual behavior, injection drug use, or both. In 2014, the U.S. Centers for Disease Control and Prevention (CDC) published comprehensive clinical practice guidelines to support the provision of PrEP [3] and in the 2015 updated National HIV Prevention Strategy, “full access to comprehensive PrEP services”^(p. 3)^ for people at risk for HIV was highlighted as 1 of 4 key areas of critical focus [4].

Despite the push for PrEP implementation by federal health authorities [3,4] and growing consumer demand [5], uptake by medical providers has been slow. Recent surveys of U.S. primary care providers and HIV specialists have generally indicated moderate-to-high awareness of PrEP but low rates of actual prescription of PrEP [6]. A number of theoretical and practical barriers to providing PrEP have been reported by providers, with the following six being especially prominent: uncertainty about financial coverage/reimbursement for PrEP-related costs (Barrier 1); logistical concerns around PrEP implementation, including the burden of ongoing laboratory testing and time required for risk and adherence counseling (Barrier 2); challenges with determining patient eligibility, including anticipated difficulty applying risk criteria and eliciting an accurate sexual history from patients (Barrier 3); concerns about patient adherence to daily medication and follow-up appointments (Barrier 4); discomfort exposing otherwise healthy individuals to potential medication side effects when other prevention options are available (Barrier 5); and apprehension about patients increasing their risk-taking behavior with PrEP use (i.e., risk compensation; Barrier 6) [7–14].

Providers who have firsthand experience prescribing PrEP are a largely unstudied population possessing critical insight that may help PrEP-inexperienced providers overcome barriers to prescription. In the current qualitative, interview-based study, we explored PrEP implementation among early-adopting U.S. providers, probing specifically for how the six commonly cited prescription barriers and others were experienced and addressed. Additionally, we sought recommendations from these early adopters about content to include in PrEP educational trainings targeting PrEP-inexperienced healthcare providers. Most of the providers interviewed were infectious disease specialists practicing in university-affiliated medical centers or hospitals in the Northeastern or Southern U.S.; thus, their insights may be especially informative for PrEP implementation and training in similar contexts and be adaptable to other healthcare and geographic settings.

## Methods

### Participants

Twenty-eight English-speaking U.S. healthcare providers with PrEP prescribing experience and/or expertise were invited to participate in the study via email or in-person outreach by the principal investigator (PI: SKC; 5 providers), email outreach by co-authors (18 providers), or email referral from other participants (5 providers). Of these, 22 responded to the invitation expressing interest and 20 were ultimately interviewed. Six providers were unresponsive to the invitation and two providers who expressed initial interest cancelled their interview appointments and were unresponsive to follow-up inquiries. Two providers with expert knowledge who participated in interviews were excluded from the current analyses because they had no direct clinical experience prescribing PrEP. The final sample included 18 providers who had previously prescribed PrEP to one or more patients. Recruitment was halted when data saturation was reached for main themes.

### Procedure

Interviews were conducted by the PI in person or by phone between September 2014 and February 2015 and lasted approximately 60–90 minutes [M(SD) = 81(10.4)]. Interviews were semi-structured, following a thematically organized guide that included lead questions and follow-up prompts. Primary themes included: PrEP experience, PrEP attitudes and prescribing intentions, patient/provider communication about sex, equitable provision of PrEP, and training experiences and recommendations. Participants were specifically asked about experiences with PrEP initiation, monitoring, and termination, including challenges encountered. Often, the six barriers described above (financial coverage, implementation logistics, etc.) emerged organically in this context, but prompts were used to generate discussion as needed. Training recommendations were primarily offered in response to the interviewer’s inquiries about specific content and strategies for a 1-hour, provider-targeted PrEP/cultural competence intervention. In addition to the interview, participants completed a brief questionnaire assessing their sociodemographic characteristics, medical background, and prior clinical experience with PrEP [see **S1 Fig**]. These self-reported characteristics were solicited to describe the sample and contextualize reported experiences. Participants were offered a $100 gift card as compensation for participation.

All study procedures were approved by Yale University’s institutional review board (Human Research Protection Program; HSC Protocol #1308012487) prior to inception. A waiver of participants’ written/signed consent for this study was requested and approved because participants’ written consent would have linked them to the study, and the principal risk of study participation was the potential harm resulting from a breach of confidentiality. Instead, verbal informed consent was obtained from all participants at the outset of the interviews. Verbal consent procedures involved the PI presenting an overview of the study, reviewing the approved verbal consent form with the participant, and inviting the participant to ask questions and look over a copy of the form prior to vocalizing consent. Providers were informed that the study aimed to explore PrEP attitudes, experiences, and perceived training needs and that data would be used to develop a provider-targeted PrEP educational intervention. Ethical considerations—including confidentiality, risks and benefits associated with participation, and participant rights—were covered when reviewing the consent form. For each participant, the PI signed her own name on a hard copy of the form, indicating she had witnessed the verbal consent, and securely stored the document. Participants were provided with a copy of the consent form for their own records, along with contact details for study personnel and Yale University’s institutional review board.

### Analysis

Interviews were audio-recorded and transcribed verbatim. Transcripts and field notes were imported into NVivo 10 to support textual data management and analysis. Analysis was guided by the Framework Method, a systematic approach to organizing and elucidating themes from textual data that has been specifically recommended for use within multidisciplinary health research [15]. This method is particularly well suited for the present study given its applicability to both inductive and deductive thematic analysis and the structure it provides to facilitate comparison within and between interviews. The Framework Method encompasses seven stages: transcription, familiarization with data, coding, development of a working analytic framework, framework application, data charting, and interpretation [15]. The PI drafted an initial analytic framework containing codes, or descriptive labels used to define concepts (e.g., *eligibility determination*), which were organized into broader conceptual categories (e.g., *PrEP experience*). The framework was subsequently refined through an iterative process, during which she and two co-authors (AIE and LAG) independently coded transcripts (i.e., applied codes to textual data) and then reconvened to discuss, revise, and add new codes. This process allowed for identification and documentation of newly emergent themes. The final multilevel framework was used by AIE and LAG to code all transcripts, with 20% overlap (double-coding) of transcripts to ensure consistency in code application. Coded text was reorganized relative to the prescription barriers and training recommendations and reviewed by the PI, employing NVivo’s matrix coding/query functions. This allowed for systematic identification of themes and points of divergence across interviews as well as selection of illustrative quotes. In the Results section that follows, illustrative quotes and other participant-specific data are presented with participant identification number, geographic setting, and number of PrEP patients (Pts.) in brackets.

Reflexivity was sought throughout the process of data collection and analysis. The PI and co-authors involved in coding entered into this research with knowledge of PrEP’s efficacy and the shared belief that PrEP should be accessible to people at risk for HIV infection. In conducting the interviews, the PI informed providers of her academic position, that she was not a medical provider, and that she had no ties to the pharmaceutical company that makes Truvada^**®**^. The PI sought to pose interview questions in a neutral manner without conveying her personal opinions. To assess the PI’s effectiveness in doing so, an *interviewer bias* code was included in the analytic framework and applied by co-authors to any interview questions in the transcripts that they perceived to have been worded non-neutrally and to have potentially influenced participant responses. In the rare instances that this code was applied, responses were reviewed and excluded as appropriate.

## Results

### Sample Characteristics

Participant characteristics are presented in **Table 1**. Providers ranged in age from 31 to 53 years [M(SD) = 43(8.3)] and were racially diverse. They were primarily non-Hispanic and male, and nearly half were sexual minorities. Most practiced in the Northeastern or Southern U.S. The most commonly reported practice settings were university-affiliated medical centers and hospitals. Nearly all participants were physicians and most identified as HIV and infectious disease specialists. Clinical experience caring for patients living with and at risk for HIV infection was common. Most participants had prescribed PrEP as part of their clinical practice, and a substantial minority had prescribed as part of a research study (e.g., demonstration project or observational study). Prior experience prescribing PrEP ranged from 2 to 325 total patients, with a median of 6 clinical practice patients among those prescribing in clinical practice (range = 2 to 56) and a median of 145 research patients among those prescribing in a research context (range = 1 to 300). All participants indicated that they were “comfortable” or “very comfortable” prescribing PrEP according to a 5-point scale ranging from “very uncomfortable” to “very comfortable.”

**Table 1:** Characteristics of Early-Adopting PrEP Provider Sample (*n* = 18).

		*n* (%)
**Age**		
	30–39	7 (38.9)
	40–49	5 (27.8)
	50–59	6 (33.3)
**Ethnicity**^**a**^		
	Latino/Hispanic	2 (11.8)
	Non-Latino/Hispanic	15 (88.2)
**Race**		
	Asian	6 (33.3)
	Black/African American	2 (11.1)
	White	7 (38.9)
	Other	3 (16.7)
**Gender**		
	Female	4 (22.2)
	Male	13 (72.2)
	Nonbinary	1 (5.6)
**Sexual Orientation**		
	Gay/Lesbian	8 (44.4)
	Heterosexual	10 (55.6)
**Education (Highest Degree)**		
	Medical Doctor (MD or MD/PhD)	17 (94.4)
	Other	1 (5.6)
**Practice Setting**^**b**^		
	Community Health Center	3 (16.7)
	Hospital	6 (33.3)
	Private Practice	1 (5.6)
	University/Academic	9 (50.0)
**Geographic Location**		
	Midwest	1 (5.6)
	Northeast	12 (66.7)
	South	4 (22.2)
	West	1 (5.6)
**Medical Role (MDs only)**^**a**^		
	HIV/Infectious Disease (ID) Specialist Only	13 (76.5)
	Primary Care Provider Only	1 (5.9)
	Both HIV/ID Specialist and Primary Care Provider	3 (17.6)
**Clinical Experience with High-Incidence Groups**^**b**^		
	Men Who Have Sex with Men	18 (100.0)
	People Who Exchange Sex for $, Drugs, etc.	17 (94.4)
	People Who Inject Drugs	18 (100.0)
	Transgender Women	18 (100.0)
**HIV Treatment Experience**		
	≥1 HIV+ Patients	17 (94.4)
	0 HIV+ Patients	1 (5.6)
**Context of Prior PrEP Prescription**^**b**^		
	Clinical Practice	17 (94.4)
	Research	7 (38.9)
**Comfort Prescribing PrEP**		
	Comfortable	4 (22.2)
	Very Comfortable	14 (77.7)

a n = 17 for these variables

b Categories not mutually exclusive

### PrEP Experiences

Most participants characterized their overall experience with PrEP positively and were enthusiastic about continuing to offer PrEP to their patients. The six barriers to PrEP prescription commonly reported by providers in earlier research (financial coverage, implementation logistics, etc.) were found to be minimal or manageable with adequate resources among our sample of early-adopting PrEP providers.

#### Barrier 1: Financial Coverage

Providers reported that most PrEP patients obtained coverage for medication costs, typically through private insurance, Medicaid, or pharmaceutical company (Gilead^**®**^) patient assistance programs. However, navigating the requirements of these funding sources was often time-consuming and effortful for healthcare teams, and could be discouraging for patients. This barrier was the most commonly cited challenge to PrEP implementation. A few providers had not experienced problems in this realm, but most reported that they or their staff had dedicated significant amounts of time to phone conversations and paperwork in order to establish and maintain coverage. One provider [P9/West/325 Pts.] described his experience appealing a rejected claim as a “*bit of a pain in the neck*,” but conceded that “*it’s never been a show stopper*” and was “*nothing out of proportion with what we see on a regular basis with insurance companies*.” However, another provider [P10/Northeast/14 Pts.] regarded the insurance requirements for PrEP as unusually onerous, recounting a claim that had been rejected by the pharmacy: *“You call up and you say*, *‘Do you need a prior authorization?’ And they say*, *‘No*.*’ And then I’ve called the insurance*, *and it turns out that I just have to talk to their pharmacist to let them know I’m an HIV doctor and I know about PrEP*. *It’s this bizarre… additional level of scrutiny that isn’t–I’ve never run across it before*.” Several providers described a paradox whereby uninsured patients could access PrEP through patient assistance programs, but some insured patients, ineligible for patient assistance programs due to their insurance status yet unable to afford their high insurance deductibles, could not access PrEP. (Of note, interviews were completed prior to Gilead^**®**^ revising the terms of their copay assistance program to allow patients to access their full annual allocation upfront vs. facing monthly restrictions, which has likely improved access for insured patients with high deductibles.)

Added costs associated with healthcare visits and laboratory testing sometimes posed problems. One provider [P7/South/47 Pts.] overcame this barrier by negotiating with the local health department to perform laboratory work at cost and directing income from his clinic’s insured patients to support the costs of uninsured patients.

#### Barrier 2: Implementation Logistics

When developing plans for patient treatment and follow-up, providers expressed sensitivity to patients’ needs and preferences and sometimes deviated from established protocols in order to accommodate them. For example, a heterosexual female PrEP patient who was trying to become pregnant expressed a preference to see her provider [P6/Northeast/4 Pts.] every month rather than every 3 months as recommended by her provider based on CDC guidelines; consequently, her provider scheduled her for monthly visits. Another provider [P14/Northeast/4 Pts.] advised a PrEP patient who reported frequent sexual activity with inconsistent condom use to return to the health center monthly to be tested for sexually transmitted infections (STIs) rather than quarterly as was standard for his other patients. One provider [P10/Northeast/14 Pts.] described tailoring plans for patients who were intermittently sexually active by prescribing daily PrEP for the anticipated period of sexual activity, plus 2 weeks prior (for male patients who have sex with men) and 4 weeks after. The provider explained, “*I have one patient whose partner lives far away*, *and they only see each other like every 6 months*. *And so why take the medication every day [while apart] when you know there is no benefit for taking that medication during that time?*”

HIV/infectious disease specialists described different models for coordinating PrEP care with primary care providers. One specialist [P11/Northeast/15 Pts.] had primary care providers refer patients to him for an initial in-depth consultation, which could last up to an hour. He felt more capable of accommodating such lengthy appointments and better prepared to answer detailed questions given his experience prescribing PrEP and familiarity with HIV medications from treating HIV-positive patients for many years. After the initial consultation, patients were followed by their primary care providers.

A flexible model of collaboration was described by a specialist [P17/Northeast/2 Pts.] in a community health center at which primary care providers and infectious disease specialists both practiced. She similarly expressed sensitivity to the time constraints of her colleagues in primary care and was willing to accept referrals from them, but was also open to simply providing support as needed if they preferred to prescribe PrEP themselves.

Providers discussed different approaches to developing PrEP care systems within their practice. This often involved cross-disciplinary collaboration and appointment of leaders. One provider [P15/Northeast/6 Pts.] described his clinic’s formation of a “*PrEP working group*,” which was *“a multidisciplinary team to figure out how we could incorporate PrEP into our practice*,*”* and his appointment as the *“PrEP champion*,*”* who served as *“the point person or provider who would lead the initiative on prescribing PrEP*.” This provider and others talked about the value of having support staff on site. These staff members could educate patients about PrEP, communicate with insurance companies, help patients complete paperwork, schedule and remind patients about appointments, and/or perform risk and adherence counseling, which alleviated provider burden.

#### Barrier 3: Eligibility Determination

Providers commonly expressed the view that determining a patient’s eligibility for PrEP was a decision that did not fall on their shoulders alone. Rather, it was seen as a decision that was shared with the patient. One provider [P5/Northeast/56 Pts.] described this joint decision-making approach as “*harm reduction*, *so*, *try to meet people where they're at*. *So*, *I really try never to tell people what to do*. *I really try to work with them to come up with a plan that's right for them*.*”* Another provider [P1/South/150 Pts.] saw his patients as having unique expertise that he as the provider lacked—firsthand knowledge about past risk behavior and intended future behavior. Therefore, he felt his patients were almost *better* positioned to determine whether PrEP was a good fit for them, stating, “*We are giving them a choice*. *We're empowering them to sort of be their own doctor*.”

When providers were asked how they determined patient eligibility, many specifically referenced the CDC guidelines or described criteria consistent with the CDC guidelines, including consideration of number, gender, and serostatus of partners as well as epidemiological context. However, providers also described scenarios where they prescribed PrEP to patients who did not meet these criteria. For example, one provider [P3/Northeast/202 Pts.] said, “*We don’t wait for someone to be at risk*. *We also offer it to them*, *like*, *‘Do you–could you see yourself*, *in the future*, *being at risk? Is this something that you could incorporate into your life?’ We don’t wait*. *Like*, *women who are on–starting birth control pills*, *often will start on the pill before they are at risk for pregnancy…I think it should be the same way [for PrEP]*.*”* Thus, for this provider, greater emphasis was placed on anticipated future behavior than on past behavior as in the CDC guidelines.

Providers expressed mixed views with respect to prescribing PrEP to the “worried well.” Some but not all prescribed in cases where the anticipated benefits of PrEP appeared to be primarily psychological. However, even providers who indicated that they would discourage a low-risk patient from initiating PrEP reported that, barring any medical contraindications such as kidney dysfunction, they would ultimately prescribe if the patient insisted.

#### Barrier 4: Adherence Concerns

Experiences with patient adherence were variable. Providers noted that adherence was particularly strong among patients who had sought PrEP (self-referred). “*Individuals who are actively seeking and using–taking PrEP right now are the extremely highly motivated*…*And these are… what we consider as healthcare providers to be ‘good patients’ because they do as we ask them to do*. *They come back on time*. *They remember their appointments*. *And they're pretty much on top of all of it*” [P14/Northeast/4 Pts.]. Another provider [P9/West/325 Pts.] corroborated this impression of strong adherence among self-referred patients in his clinical practice, estimating that 80% took PrEP “*with incredible anal retentive fidelity*”; however, he described the remainder as being overwhelmed by the follow-up requirements and “*disappearing right away*.”

A few providers commented that one of the target demographics for PrEP—young men who have sex with men—were often unaccustomed to taking pills or going to medical appointments, and sometimes needed added supports. One provider [P5/Northeast/56 Pts.] estimated that about 30% of his younger PrEP patients missed follow-up appointments, which he attributed to forgetting. Another [P7/South/47 Pts.] noted that the lack of a daily routine among this group contributed to adherence problems, stating, “*More than half of my patients who are younger than 25 years old–there’s not a single thing in their day that they do at the same time… Many of our patients… don’t know how to even create any degree of structure*.”

#### Barrier 5: Side Effects

Providers generally reported that their patients had experienced minimal side effects, with several providers who had fewer (under 10) PrEP patients even reporting no adverse effects. Other providers observed a start-up syndrome involving nausea and/or headaches in a subset of their patients in the first 2 to 4 weeks. More serious symptoms were rare; one provider reported stopping PrEP when a patient exhibited a change in kidney function, which reversed after discontinuation. Providers reported that a small minority of patients opted to discontinue PrEP due to nausea, headaches, or other symptoms (e.g., rash, fatigue), but sometimes offered caveats concerning symptom attribution, such as “*Some of the times*, *in my opinion*, *it wasn’t related to the medication*, *but they were convinced it was*” [P2/South/147 Pts.].

#### Barrier 6: Anticipated Behavior Change (Risk Compensation)

Providers indicated that most of their patients reported no change in condom use after initiating PrEP. This was especially the case among patients at the two extremes of the condom use spectrum—those who never used condoms and those who always used condoms prior to PrEP initiation. As stated by the provider reporting the highest number of PrEP patients [P9/West/325 Pts.], “*We’re certainly not seeing people who are having a hallelujah risk compensation effect*.”

Providers reported both decreases and increases in risk behavior among subsets of their patients. Providers who observed decreased risk behavior attributed the change to patients being more engaged and empowered in caring for their health. One provider [P5/Northeast/56 Pts.] indicated that his patient described it as a matter of accountability, stating, “*So just knowing he had to see me every 3 months and that I was going to ask him questions like ‘How many people have you had sex with? Are you still using condoms?’ He felt like he was a little more accountable to me and therefore was paying a little more attention to his own health*.”

In the select instances where providers did see increased risk-taking, they reported responding by engaging those patients in supportive, nonjudgmental conversations, reiterating that PrEP in combination with condoms offered more protection than PrEP alone and that condoms were important for protection against other STIs. The providers did not see increased risk behavior as grounds to discontinue PrEP.

#### Other Barriers

In addition to addressing the six anticipated barriers to PrEP provision, providers identified other challenges related to accessing and engaging priority populations. From the perspective of one infectious disease specialist [P17/Northeast/2 Pts.], “*Either the community isn’t aware enough about it or their primary care providers are still not talking about it enough*, *are not confident enough*, *[or] are not able to answer their questions*, *so that there’s a gate and the gate is really closed off before people actually get to me*.” Participants perceived additional factors to be interfering with PrEP provision and referral by other providers, including discomfort discussing sexuality and disapproval of patient motivations for seeking PrEP. One participant [P1/South/150 Pts.] believed that provider conceptions of PrEP as a “*a gay man’s prevention tool*” limited PrEP education and provision/referral for patients belonging to other social groups. Participants perceived several barriers to individuals at risk actively seeking PrEP from providers, including lack of PrEP awareness, medical mistrust, absence of existing ties to the medical system, and structural hurdles (e.g., health centers’ daily hours of operation failing to accommodate those with less work flexibility).

### Training Recommendations

Nearly all providers indicated that they were self-educated with respect to PrEP. Rather than participating in formal training, their knowledge about PrEP was obtained through a combination of reading relevant literature, attending professional talks and conferences, consulting with colleagues, and treating HIV-positive patients with the same antiretroviral medication (Truvada^**®**^). As early adopters of PrEP, many of the providers were PrEP educators themselves, leading formal trainings for other providers. They offered a wide range of suggestions regarding content, strategy, and logistics surrounding PrEP training, which are summarized in **Table 2**. Sexual history-taking and sexual minority competence were among the topics most commonly raised as training priorities based on the perceived learning needs of other providers. Several participants expressed concern that provider-targeted PrEP trainings were not being widely implemented, with one provider [P9/West/325 Pts.] commenting, “*Unless we get a lot more providers trained in how to do this*, *it is only gonna be well-resourced people who have access to [PrEP]*.”

**Table 2:** Recommendations for Provider-Targeted PrEP Training Programs.

**A.**	**Topics/Content To Cover During Training**^**a**^	**Description**	**Illustrative Quotes**
	PrEP Background/ Clinical Protocol	Review PrEP medication history, dosing, and side effects; clinical trial evidence; CDC guidelines for eligibility, initiation, and management; and barriers to provision (+ solutions)	*If you talk to primary care doctors*, *you need to talk probably a fair amount about the medicine and about the follow-up and monitoring procedures*. *So*, *what is Truvada? Some of these docs may never have heard of it before*. *So*, *doing this has been out for a while*, *we use it to treat HIV*, *we have a lot of experience with it*, *FDA approved it for HIV-negative people*, *here are the side effects we worry about*, *here's what you need to watch out for*, *here's the dose*.* *.* *. [P5/Northeast/56 Pts.]
			*I think if you can somehow get a quick summary of kind of what got PrEP to be an intervention as far as a little bit about the studies and a little bit about how the guidelines came about and what the kinda gist of the guidelines is and maybe talk about some of the barriers*. [P6/Northeast/4 Pts.]
			*I think being very concrete on the mechanistic operations*, *the operational details of how you do it*, *what assessments are done*, *at what time points*, *and what happens at each visit are important because I think that’s one of the things that gives people the most anxiety is not knowing what is gonna happen at every visit*. [P9/West/325 Pts.]
	Sexual History-Taking	Improve providers' comfort discussing sex with patients and proficiency doing so in a non-judgmental way	*Sexual history-taking*. *If people never even start with that*, *they never–they’re never gonna get ‘round to prescribing PrEP*, *and it seems like that should be so straightforward*, *but it’s not*. *People really aren’t trained*. [P3/Northeast/202 Pts.]
			*They're gonna have to practice and feel very comfortable*, *you know*, *asking about all these [sex-related] questions and still make it very professional*, *not be judgmental*, *make sure that*, *you know*, *patients are not seeing that you're just*, *you know*, *making faces when they're saying things*, *you know what I mean? Just make sure that they*.* *.* *. *have ears for everything that the patient has to say*. *So that should be a very important part of your [training]*, *you know*, *how to take a sexual history*, *how to feel comfortable doing it*. [P12/South/30 Pts.]
	Sexual Minority Competence	Enhance understanding of and competence discussing sexual behavior and sexual health with sexual minority patients	*There has to be intense training on sexual minority practices or just the prevalence of how many sexual minorities are in your practice*. *People don’t even know*. *People never ask the question*, *"Do you have sex with men*, *women*, *or both?"*.* *.* *. *If you don’t know what MSM is and you don’t know how to interact and you are not culturally competent to even have a comfortable conversation that makes them comfortable*, *I don’t know how you are supposed to distribute PrEP*. [P13/Midwest/8 Pts.]
			*Even just when you're talking to a gay man and you use the terms "top" and "bottom"… I think if you are able to use the jargon*, *then–and understand the jargon–then patients feel a lot more comfortable talking about certain things*. [P4/Northeast/4 Pts.]
	Shared Medical Decision-Making	Emphasize the need for a patient-centered/ collaborative approach to PrEP care	*It’s a very different mindset than providing care for a chronic disease*. [P9/West/325 Pts.]
			*For HIV prevention*, *there's less of "Here's the problem and here is what you need to do to fix it”… And so there is a lot more shared medical decision-making… It's more kind of like “What do you want to do? What do you think is right for you with prevention?” And it's not like—there's not sort of a formula [for PrEP] necessarily where you're prescribing something as a result of a specific disease condition…there's not a right answer*. [P4/Northeast/4 Pts.]
	HIV Epidemiology	Familiarize providers with the sociodemographic groups considered to be at highest risk for HIV	*HIV is disproportionately people of color*, *and lower socioeconomic backgrounds*, *and so not to*.* *.* *. *recognize that there are racial differences in the disease and who is at risk is a problem*. [P10/Northeast/14 Pts.]
			*I give a 15-minute talk at provider meetings for–that largely are the community-based health centers and my first 1–2 minutes are on "Here is the epidemiology in a nutshell*.*" I would–I can’t count the number of times that people are like*, *"Oh my god*, *that’s what it is?!" "We haven’t changed our rates over 10 years*.*" "Oh my god*.*" "Oh*, *it’s like 63 percent*.* *.* *. *MSM*.*" "Oh*, *I didn't know that*, *I thought it was IV drug use*.*"*.* *.* *.*The cart has been going without the horse*. *You need the horse first*, *which is epidemiology and sexual minority health*. [P13/Midwest/8 Pts.]
**B.**	**Strategies for Training Delivery**	**Description**	**Illustrative Quotes**
	Empathize with the Audience	Communicate understanding of time limitations and other implementation barriers	*If anything is even acknowledged about how*, *kind of overstretched the clinician is today and that kind of gets acknowledged up front*, *they're immediately gonna start listening*.* *.* *. *'cause it's like*, *oh*, *this person gets that like–they get the milieu I'm in*. [P6/Northeast/4 Pts.]
	Be Concise and Direct	Present main message and essential information in a clear and up-front manner, as providers may have limited time, interest, and/or attention	*The way the medical system is set up now is everything's bam bam bam and doctors are short on time*. *So*, *you're going to have limited time to engage these doctors*. *So*, *you want the most bang for your buck*.* *.* *. *I would just think about*, *you know*, *cut to the meat*. *When I personally go to these lectures–I know a lot of my colleagues feel the same way–You want to know what you need to know*. [P5/Northeast/56 Pts.]
			*It has to be something really quick and effective*, *because in order to do anything more structured*, *you're gonna have to select a group of providers who are very motivated*. [P7/South/47 Pts.]
	Emphasize Feasibility	Stress the ease of implementation and simplicity relative to other forms of medical care already being practiced	*What you have to do is you have to compare PrEP to an existing primary care issue*, *which would be like anti-coagulation or diabetes or even hypertension*, *and how easy the monitoring is and how nontoxic this medication is*.^b^ [P13/Midwest/8 Pts.]
			*People liked the birth control analogy… It's something that they can relate to*. [P14/Northeast/4 Pts.]
			*The other thing that's probably helpful is sort of highlighting the ease of prescribing PrEP*.* *.* *.*so the perception doesn't come across as this is a really onerous process*. *'Cause I think anything that starts coming across as this is gonna take too much time and this is gonna be really involved*, *it's gonna be like*, *you know*, *I'm not really gonna do this*. [P14/Northeast/4 Pts.]
	Reframe Sexual Health (and PrEP) as Being Within Providers' Purview^c^	Help providers recognize that sexual health is part of their responsibility	*You have to educate [primary care providers] on that*, *saying*, *like*, *"You're just not here to take care of diabetes*.* *.* *.* *.*they're your patients*, *you have to take care of them as a whole*.* *.* *. *you have to be proficient in everything that you do with your patient*, *and if you are taking care of your patient as a whole*, *you wanna make sure that you also include those questions–their sexual history*.*"* [P12/South/30 Pts.]
	Apply to Real-World Patient Cases	Integrate instructional information with medical case example(s)	*Clinicians like cases*, *so when I give talks I usually*.* *.* *. *present a case*.* *.* *. *You can keep going back to the case during your talk*, *and if there are barriers that you wanna illustrate*, *you can show the–"Well*, *then back to our patient and the doctor*. *Then the patient said*, *'I don't have health insurance*,*‴ you know*, *stuff like that*. *It kinda keeps me engaged 'cause I like talking around cases*, *and I think the clinicians*, *you keep pulling them back in from kind of what can be a little bit dry from the literature and stuff*. [P6/Northeast/4 Pts.]
	Tailor Content to Location and Audience	HIV epidemiology, financial coverage/reimbursement, and patient preferences may vary by geographic region, and provider learning needs will vary by provider type; education should be tailored accordingly	*So we’ve worked a lot with groups of young African American MSM in [city name] to learn from them what they think the messages need to be and how these services need to be delivered*. *So we’ve gone straight to the sources for training about how to do it in a way that is culturally appropriate*, *acceptable and even desirable*.* *.* *. [P9/West/325 Pts.]
			*You really have to take a step back and say*, *who are you going to target it to? So if you're going to target it to ID doctors*, *it's going to be a very different program than if you're going to target to primary care doctors*. [P5/Northeast/56 Pts.]
	Provide Concrete Examples	Model and/or script communication with a patient, including specific language	*You can talk about things that are really abstract*, *but it's important to model the behavior*. *You can't tell someone*, *"You talk to a patient respectfully*,*" when they have never seen the difference between one and the other*. [P7/South/47 Pts.]
			*Giving people concrete strategies*, *'cause there are a lot of guidelines and things that say very vague things*, *like you know*, *"You should obtain a sexual history in a non-judgmental way*.*" But*, *for I think most people*, *that doesn't help*, *because it's kind of like*, *"Well*, *how do I operationalize that?"* [P14/Northeast/4 Pts.]
			*Scripts are helpful*. *You know*, *how to go about asking the questions*.* *.* *. *about sex or about having a conversation about HIV prevention*. *You know*, *like if I’m going to prescribe PrEP*, *what should I say about condom use? I always feel like people like to know how to say it*. [P17/Northeast/2 Pts.]
	Incorporate Data	Support assertions with empirical evidence	*Studies are what we use*. *That's what we base most of our judgments on*. [P2/South/147 Pts.]
	Offer Supplemental Resources	Provide web links, contact information, and other resources to support further PrEP education and uptake	*I think that [the training] might work if it is 1 hour*, *but maybe just give them the opportunity–like*, *if they're very interested and to make sure that they can get more*. [P12/South/30 Pts.]
			*Somebody that is there to help me…*.*the PrEP hotline*, *somebody in the community*.* *.* *. *that they know who they can call if they feel like there is some dilemma*. [P17/Northeast/2 Pts.]
**C.**	**Training Logistics**	**Description**	**Illustrative Quotes**
	Modality	Web delivery is generally convenient and well received by providers, but less optimal than in-person delivery given its vulnerability to distraction and prohibition of group discussion	*I know web-based learning is very popular because it’s less expensive*, *people don’t have to travel*, *but the problem is people multitask and they don’t pay attention*.* *.* *. *I know what I do and when I have to watch a webinar*, *I’m also answering email*, *I’m also signing forms*, *I’m also doing other things and that’s just the reality*. *Unless you make me stop everything else that I’m doing and focus*, *there are too many other demands on people’s attention spans*. [P9/West/325 Pts.]
	Audience Participation	Audience response technology and medical case discussions tend to be universally well received, whereas audience role-playing and group break-out sessions may be met with mixed or negative reactions	*I mean I think role-playing is always helpful*, *even if you see people who do it and you think*, *that’s probably not the way I would do it*. *It helps you think that through before you’re in the situation*. *If you just look*, *you know*, *at a slide that says*, *here are the CDC criteria*, *that doesn’t get you to the encounter*. [P17/Northeast/2 Pts.]
			*I think that role-playing is something we did through medical school and training*, *but I think that even now–I'm 3 years out of my last training–I'd find it too juvenile*. [P5/Northeast/56 Pts.]
			*I mean an audience response would be another way to do it*, *where you get*. *people to anonymously push a button*. *That’s a fun way*. [P10/Northeast/14 Pts.]
	Trainer Credentials	Training directed at physicians that is led or co-led by a PrEP-experienced physician could help to generate buy-in among other physicians and ensure unanticipated clinical questions can be adequately addressed	*I think the key for endorsement of the physicians and buy-in that you need is that there is a physician sort of role model that's endorsing it… Physicians really–you know*, *they listen to other physicians*. [P6/Northeast/4 Pts.]
	* *		*There's always different medical questions that come up*.* *.* *. *you probably want an MD*, *and then talk 1-on-1 to whatever doctor that raises a question and provide more medical perspective*. *But*, *that being said*, *if you're well-versed in the medical*, *clinical care of someone on PrEP and Truvada*, *then you could probably do fine*. *It would just be like*, *what if someone asked you–it's some of the lingo too*. [P5/Northeast/56 Pts.]

^a^Some of the topics may not need to be covered (or covered in the same depth) among HIV specialists vs. other provider audiences given their respective training and experience

^b^This provider also noted that the trainer may need to clarify misconceptions about PrEP's toxicity based on non-HIV providers' knowledge of the adverse effects associated with earlier antiretroviral medications

^c^This recommendation was offered by a single provider; all other recommendations were suggested by multiple providers

## Discussion

To our knowledge, this study is the first published, in-depth qualitative examination of early-adopting healthcare providers’ experiences providing PrEP. Overall, providers described their experience of initiating and monitoring patients on PrEP favorably and were keen to continue prescribing PrEP. They described various models of implementation within and between health centers, including collaboration between infectious disease specialists and primary care providers.

The primary challenge reported was navigating payment for PrEP and associated professional services through insurance and patient assistance programs (i.e., Barrier 1). It is plausible that, over time, administrative demands and complications will lessen, medication costs will decrease, and new funding sources will emerge. Furthermore, as provider uptake increases and regional networks of PrEP providers develop, more experienced PrEP providers could potentially assist new PrEP providers in navigating local systems to facilitate financial access. Nonetheless, even in the current circumstances, providers were able to overcome financial and other obstacles to prescribing PrEP most of the time. Thus, the six commonly cited barriers were present but manageable (Barriers 1–4) or minimally present (Barriers 5 and 6). Additional challenges were noted with respect to accessing and engaging individuals who were at high risk for HIV acquisition and not currently in care, suggesting a need to scale-up outreach and adjust current health systems to more effectively meet their needs.

While the current CDC clinical guidelines are a useful starting point for identifying PrEP candidates and developing a treatment plan, PrEP providers in this study described instances when they deviated from the CDC eligibility criteria or follow-up protocol in an effort to better serve their patients. A recent cohort study reporting that only 61% of men who seroconverted on study would have met the CDC’s PrEP eligibility criteria at the preceding study visit [16] corroborates the importance of taking into consideration other factors in approaching PrEP provision beyond those listed in the CDC guidelines.

As communicated by study participants, PrEP provision is distinct from other forms of medical care in which symptoms and treatment course are clearly defined and providers are the designated experts within the patient-provider dyad. Assessment of the appropriateness of PrEP for a given patient may be optimized via a shared decision-making approach, whereby the provider contributes expertise about the medical aspects of PrEP and the patient contributes expertise about his or her behavioral history, intentions, goals, and capacity to integrate PrEP into his or her daily life. In a recent national survey of over 400 infectious disease physicians, this shared approach to PrEP decision-making was preferred over either a patient-directed or provider-directed approach, suggesting its wider acceptability as a model of PrEP provision to be promoted among providers [17]. Ultimately, PrEP prescription is contingent upon both provider capacity and patient interest and may be an ongoing conversation as patients’ life circumstances and health preferences change over time [5].

Providers offered a number of training recommendations, with particular emphasis placed on the need for increasing their peers’ comfort and competence taking culturally sensitive sexual histories. This is consistent with a recent review of providers’ preparedness to implement PrEP, which called for expanding medical curricula and skills-based training on sexual health communication to promote nonjudgmental history-taking with sexually diverse patients [6]. For motivated providers, multiple resources exist to support self-guided learning on this topic through webinars, textbooks, and other methods (see www.lgbthealtheducation.org). However, integrating content about sexual history-taking and sexual minority competence directly into PrEP training programs would extend its reach, potentially increasing the likelihood that people at risk for HIV are identified and offered PrEP as well as enhancing the comfort of both providers and patients during PrEP-related medical visits. Other PrEP training content recommended by providers in our study included clinical evidence, initiation and monitoring procedures, medication dosing and side effects, and HIV epidemiology; providers noted that training content should be tailored according to audience members’ medical backgrounds and geographic location. Taken together, these content recommendations may be useful in the development of curricula and evaluation standards for PrEP-related medical education.

Study participants also offered suggestions related to the delivery and logistics of PrEP training. Strategies for engaging providers included empathizing with their workload and emphasizing the feasibility of integrating PrEP within their current practice. Participants suggested that the ease and relevance of PrEP provision could be illuminated by comparing it to similar or more complex medical issues already within their purview (e.g., diabetes, contraception). Participants were generally enthusiastic about integrating audience response technology and medical case discussions into trainings, but expressed mixed views about including audience role-play and group break-out sessions.

Many of participants’ recommendations were rooted in their own experiences as PrEP educators, lending preliminary support for the effectiveness of these recommendations. However, the proposed content and techniques could be further refined by systematically evaluating them in the context of actual training and obtaining feedback from providers who do not prescribe PrEP. Such input, as well as input from current and prospective PrEP patients, could help to identify additional topics to cover and barriers to address. Given that providers in this study were early adopters, their reported experiences were with early-adopting patients, most of whom were men who have sex with men; new challenges to PrEP provision may emerge with patients who are later adopters or belong to other social groups, suggesting the benefit of ongoing research with both patients and providers to update and improve trainings. Programs will also need to incorporate new forms of biomedical prevention as they become available.

It is important to consider that most participants in this study were HIV/infectious disease specialists from the Northeastern and Southern U.S., and recruitment involved specific professional networks. Thus, while many of the participants’ experiences may be relevant and informative for other provider populations, they are not intended to be universally applicable across healthcare and geographic settings. As PrEP is increasingly adopted into primary care practices and other medical contexts throughout the U.S., it will be essential to understand the unique challenges experienced in these various settings so that training programs and support resources may be tailored accordingly. Another consideration with respect to our sample is that providers with more positive PrEP attitudes or favorable PrEP experiences may have been more inclined to participate in our study, reducing the likelihood that adverse PrEP experiences were captured in their personal accounts.

The CDC estimates that approximately 1.2 million U.S. adults could benefit from PrEP [18]. To date, research has documented substantial interest in PrEP among priority populations, including men who have sex with men, people who inject drugs, and heterosexual African Americans [19–21]. Members of these groups have expressed openness to learning about and/or receiving PrEP from a variety of healthcare providers, including primary care physicians, HIV specialists, HIV/STI testing counselors, psychiatrists, substance use treatment providers, emergency room practitioners, and family planning clinicians [22, 23]. Thus, PrEP has a potential role in the practice of service providers across the care spectrum. Maximizing PrEP awareness and access among individuals who stand to benefit the most will necessitate widespread provider education to ensure the capacity for PrEP provision or referral at any point of patient contact within the healthcare system. The insights shared by early-adopting PrEP providers may offer solutions to perceived barriers to provision, quell undue concerns, and establish norms that promote implementation throughout the healthcare provider community.

## Supporting Information

S1 Fig Background Questionnaire. (DOCX)
