# Supplementary material for: Putting PrEP into Practice: Lessons Learned from Early-Adopting U.S. Providers’ Firsthand Experiences Providing HIV Pre-Exposure Prophylaxis and Associated Care
Source: PLoS One. 2016 Jun 15;11(6):e0157324. doi: 10.1371/journal.pone.0157324 (PMC4909282; doi:10.1371/journal.pone.0157324)
Supplement: S1 Fig — (DOCX) [file pone.0157324.s001.docx]

*S1 Fig.* *Background Questionnaire.*

**Background Questionnaire**

Thank you for taking the time to participate in this interview! Please complete the following background information.

1. **How old are you?**

__ __ years old

(insert #)

1. **How would you describe your gender?**

- Female
- Male
- Male-to-female transgender (biologically assigned male, but identify as a woman)
- Female-to-male transgender (biologically assigned female, but identify as a man)
- Gender queer
- Other (Please specify: _____________________)

1. **Do you identify as Latino or Hispanic?**

- Yes (Please specify Latino/Hispanic ethnicity, such as “Mexican,” “Puerto

Rican,” etc.: _________________)

- No

1. **Which of the following best describes your race?**

- American Indian or Alaska Native
- Asian
- Black/African American
- Native Hawaiian/Other Pacific Islander
- White
- Other (Please specify: _____________________)

1. **In which country were you born?**
   - USA
   - Other (Please specify: _____________________)
2. **If** **you answered “Other” to Item 5: How many years have you been in the USA?** *Skip this item if you answered “USA” to Item 5.*

__ __years

(insert #)

1. **How would you describe your sexual orientation?**

- Homosexual (lesbian or gay)
- Bisexual
- Heterosexual (straight)
- Other (Please specify: _________________)
- I prefer not to say

**Questions 8-15 have to do with your medical background and perspective as a provider.**

1. **Which of the following professional degrees do you hold?**
   - Doctor of medicine (MD)
   - Doctor of osteopathic medicine (DO)
   - Physician assistant (PA)
   - Advanced practice registered nurse (APRN)
   - Nurse practitioner (NP)
   - Other (Please specify: _________________)
2. **How many years of clinical practice have you completed since finishing your education?**

__ __ years of practice

(insert #)

1. **In which of the following settings do you work?**
   - Community health center
   - Hospital
   - Physician practice group
   - AIDS service organization
   - Health department
   - University/academic
   - Corporation
   - Other (Please specify: _________________)
2. **Are you a primary care provider or specialist?**

- Primary care provider
- Specialist (Please specify your specialty: ___________________)
- Both (Please specify your specialty: ___________________)
- Neither

1. **Do you consider yourself to be an HIV specialist?**

- Yes
- No

1. **Have you ever prescribed antiretroviral medications for people who are HIV-positive?**
   - Yes
   - No
2. **Approximately how many HIV-positive patients have you ever treated?**

__ __ __ patients

(insert #)

1. **Please indicate whether any of your current or former patients have been members of the following groups (to the best of your knowledge):**

- Men who have sex with men (e.g., gay and bisexual men)
- Transgender women (biologically assigned male but identify as women)
- People who exchange sex for money, drugs, or other goods
- People who inject drugs

1. **How comfortable would you be talking to the following types of patients about their sexual behavior?** *Please circle a number to the right of each type of patient using the following key:*

- 1. A Black male patient who you believe is **1 2 3 4 5**

heterosexual/straight (has sex with women).

- 1. A Black male patient who you believe is **1 2 3 4 5**

gay or bisexual (has sex with men).

- 1. A Black female patient who you believe **1 2 3 4 5**

is heterosexual/straight (has sex with men).

- 1. A Black female patient who you believe **1 2 3 4 5**

is a lesbian or bisexual (has sex with women).

- 1. A White male patient who you believe is **1 2 3 4 5**

heterosexual/straight (has sex with women).

- 1. A White male patient who you believe is **1 2 3 4 5**

gay or bisexual (has sex with men).

- 1. A White female patient who you believe **1 2 3 4 5**

is heterosexual/straight (has sex with men).

- 1. A White female patient who you believe **1 2 3 4 5**

is a lesbian or bisexual (has sex with women).

**Questions 17-20 have to do with your experience with HIV *pre*-exposure prophylaxis (PrEP), or once daily dosing of tenofovir disoproxil fumarate with emtricitabine [TDF/FTC; Truvada®] to prevent HIV-uninfected individuals from becoming HIV-positive.**

Note: PrEP is different from *post*-exposure prophylaxis, or PEP, which is taken by HIV-uninfected individuals for a 28-day period immediately following an incident during which exposure to the HIV virus potentially occurred.

1. **Have you ever heard of HIV *pre*-exposure prophylaxis (PrEP)?**

- Yes
- No

****If** **you answered “No” to Item 17, please skip the remaining items on this questionnaire.**

****If** **you answered “Yes” to Item 17, please proceed with the remaining items on this questionnaire.**

1. **How comfortable are you prescribing PrEP to a patient?**
   - Very Uncomfortable
   - Uncomfortable
   - Neither Comfortable nor Uncomfortable
   - Comfortable
   - Very Comfortable
2. **How many HIV-uninfected patients have you PRESCRIBED PrEP to?**

a. As part of a clinical trial:

__ __ __ patients

(insert #)

b. As part of your clinical practice:

__ __ __ patients

(insert #)

1. **How many HIV-uninfected patients have you DISCUSSED PrEP with (including patients who chose not to use it)?**

a. As part of a clinical trial:

__ __ __ patients

(insert #)

b. As part of your clinical practice:

__ __ __ patients

(insert #)

**Thank you for completing this questionnaire!**
